# Pembrolizumab-Induced Sarcoid-Like Reaction: FDG-PET Scan Interpretation in the Era of Immunotherapy

**DOI:** 10.7759/cureus.9449

**Published:** 2020-07-28

**Authors:** Shiva Malaty, Craig M Bastian, Ines Ramirez-Cibes, Mahmood Shahlapour, Wishwdeep Dhillon

**Affiliations:** 1 Internal Medicine, HonorHealth Medical Center, Scottsdale, USA; 2 Hematology and Oncology, Arizona College of Osteopathic Medicine, Glendale, USA; 3 Internal Medicine, Dignity Health, Chandler, USA; 4 Oncology, Virginia G. Piper Cancer Care Network, Gilbert, USA

**Keywords:** sarcoid, cancer immunotherapy

## Abstract

Immunotherapy has revolutionized the treatment of malignant melanomas. Immunotherapy is associated with multi-system toxicities, which are referred to as immune-related adverse events (irAEs). Positron emission tomography (PET) with fluorodeoxyglucose (FDG) and CT is the preferred imaging modality to monitor disease progression in melanoma. FDG uptake by a sarcoid-like reaction (SLR) can mimic cancer progression, thereby posing a diagnostic and therapeutic dilemma. We present the case of a 39-year-old patient with malignant melanoma on immunotherapy who presented with PET scan findings of adenopathy with increased uptake. This case highlights the challenges in interpreting PET scan in the setting of an SLR.

## Introduction

Pembrolizumab is one of the first-line options for the treatment of metastatic malignant melanoma. It is an immunoglobulin G4 (IgG4) isotype anti-programmed cell death 1 (PD-1) monoclonal antibody, which leads to immune-mediated destruction of cancer cells [1]. The immune-related adverse events (irAEs) of immunotherapy commonly affect the skin, gastrointestinal (GI), and endocrine systems [2]. Positron emission tomography (PET) with fluorodeoxyglucose (FDG) and CT is known to be the most sensitive and practical imaging study to monitor disease progression in melanoma [3,4]. The activation of sarcoidosis or sarcoid-like reaction (SLR) is being recognized as an important irAE [5]. Sarcoidosis is a granulomatous disease characterized by an immune response to unknown antigens. SLRs refer to localized clinical features that do not meet all the criteria for the diagnosis of sarcoidosis [5,6]. The development of SLR during the treatment of melanoma may present a diagnostic dilemma because both SLR and malignancy are associated with FDG-avid lesions. Therefore, distinguishing SLR from malignancy is of utmost clinical relevance [7,8].

## Case presentation

**Figure 1:**
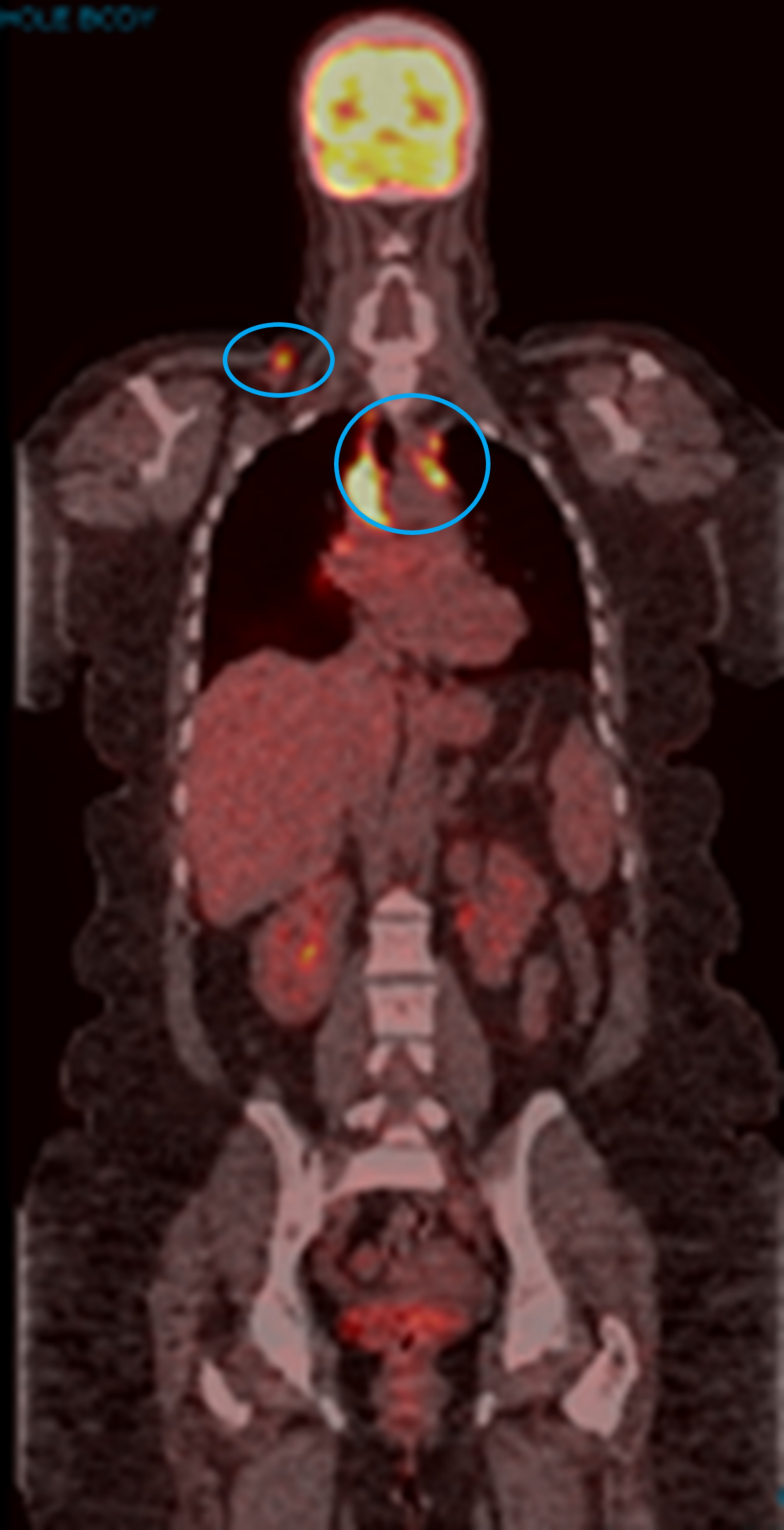
PET/CT scan in February 2017 The image shows FDG-avid lymphadenopathy in the right cervical, supraclavicular, mediastinal, and bilateral hilar regions (blue circles) PET: positron emission tomography; CT: computed tomography; FDG: fluorodeoxyglucose

A 39-year-old female patient presented to the oncology clinic with cervical lymphadenopathy first noted in January 2017. A CT scan of the neck in February 2017 showed three enlarged right cervical lymph nodes measuring up to 2.8 cm in the right supraclavicular fossa. A core biopsy of an enlarged cervical lymph node showed malignant melanoma, positive for BRAF V600E mutation. PET/CT scan showed FDG-avid lymphadenopathy in the right cervical, supraclavicular, mediastinal, and bilateral hilar regions, measuring up to 4 cm, with a maximum standardized uptake value (SUVmax) of 13.3 (Figure 1).

**Figure 2:**
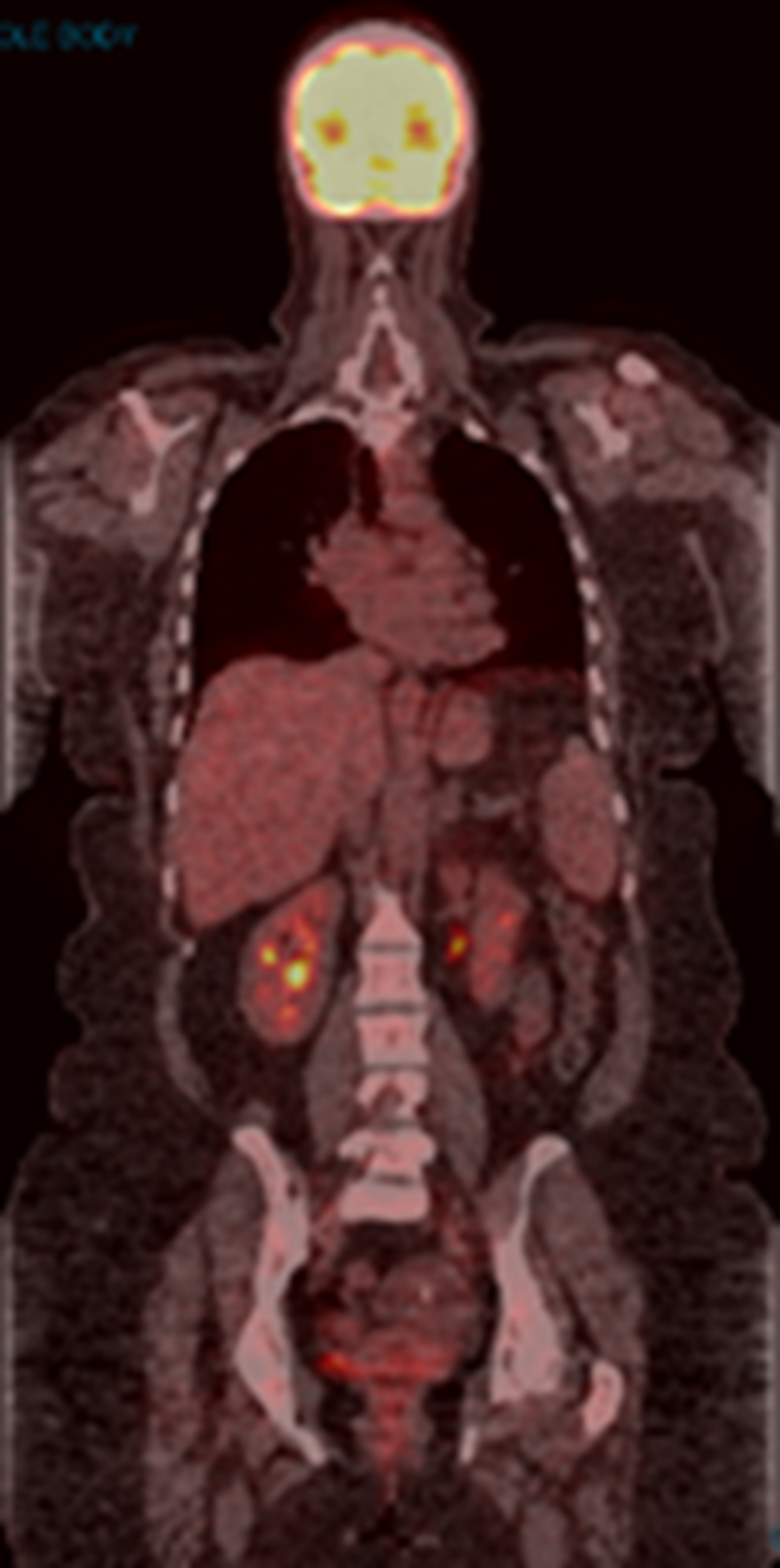
PET/CT scan in May 2017 The image shows the complete resolution of all FDG-avid lymphadenopathy PET: positron emission tomography; CT: computed tomography; FDG: fluorodeoxyglucose

**Figure 3:**
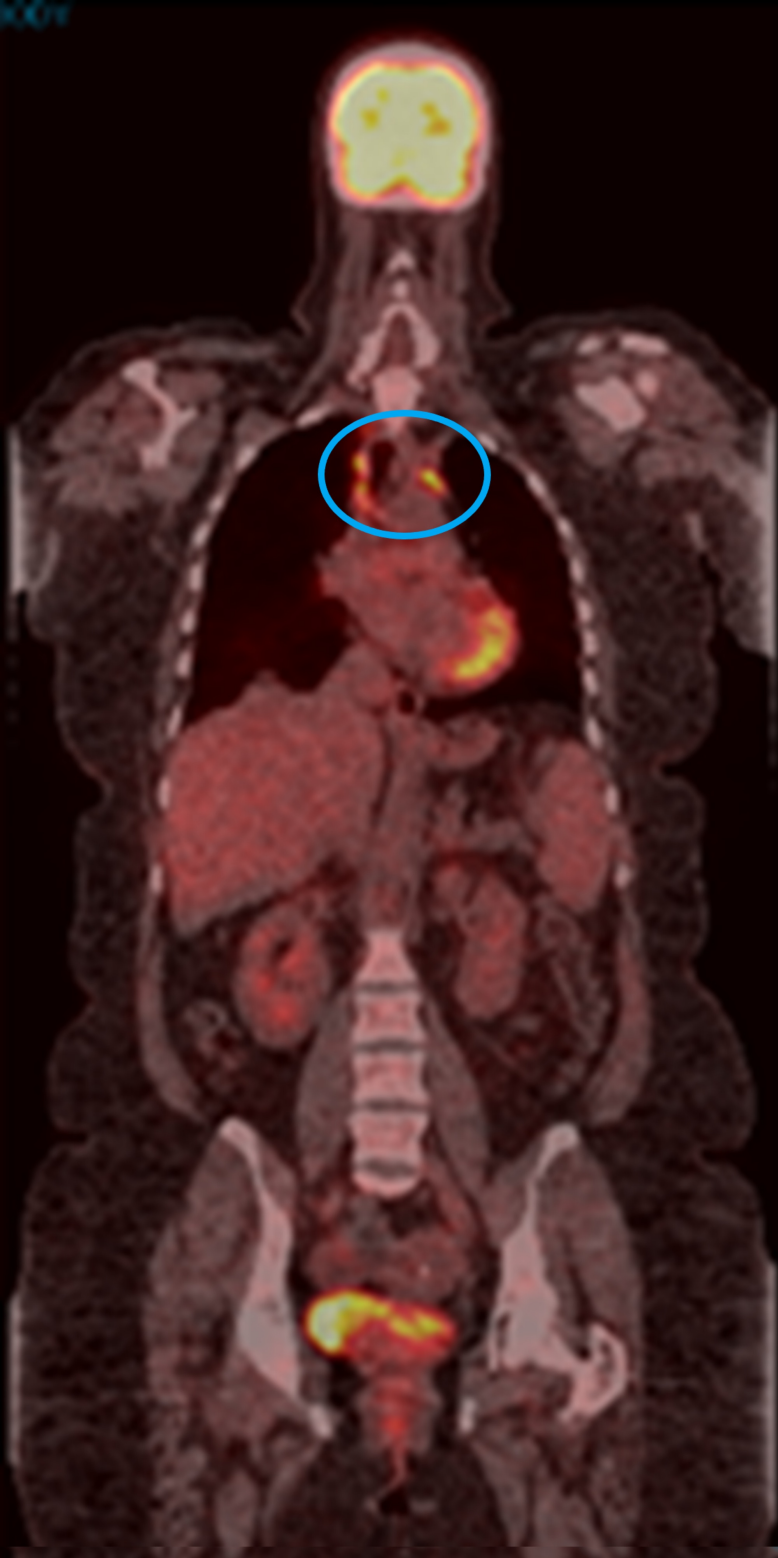
PET/CT scan in September 2017 The image shows uptake of mediastinal lymphadenopathy (blue circle) PET: positron emission tomography; CT: computed tomography

Brain MRI was negative for intracranial metastasis. Pembrolizumab 200 mg IV was initiated and administered every three weeks in March 2017. Treatment was complicated by grade 1 skin rash, joint stiffness, vitiligo, and grade 2 hepatotoxicity, managed with a brief interruption of pembrolizumab and a six-week course of tapering prednisone dose. PET/CT scan in May 2017 showed a complete resolution of all FDG-avid lymphadenopathy (Figure 2). However, repeat PET/CT scans beginning in September 2017 revealed increased uptake in various nodes of the mediastinum (Figure 3). Treatment with pembrolizumab was continued. PET CT scan in March 2018 showed persistent mediastinal lymphadenopathy with a subcarinal lymph node measuring 1.5 cm with SUVmax 7.9; there was persistent stable uptake in the periportal region with SUVmax 5.4.

**Figure 4:**
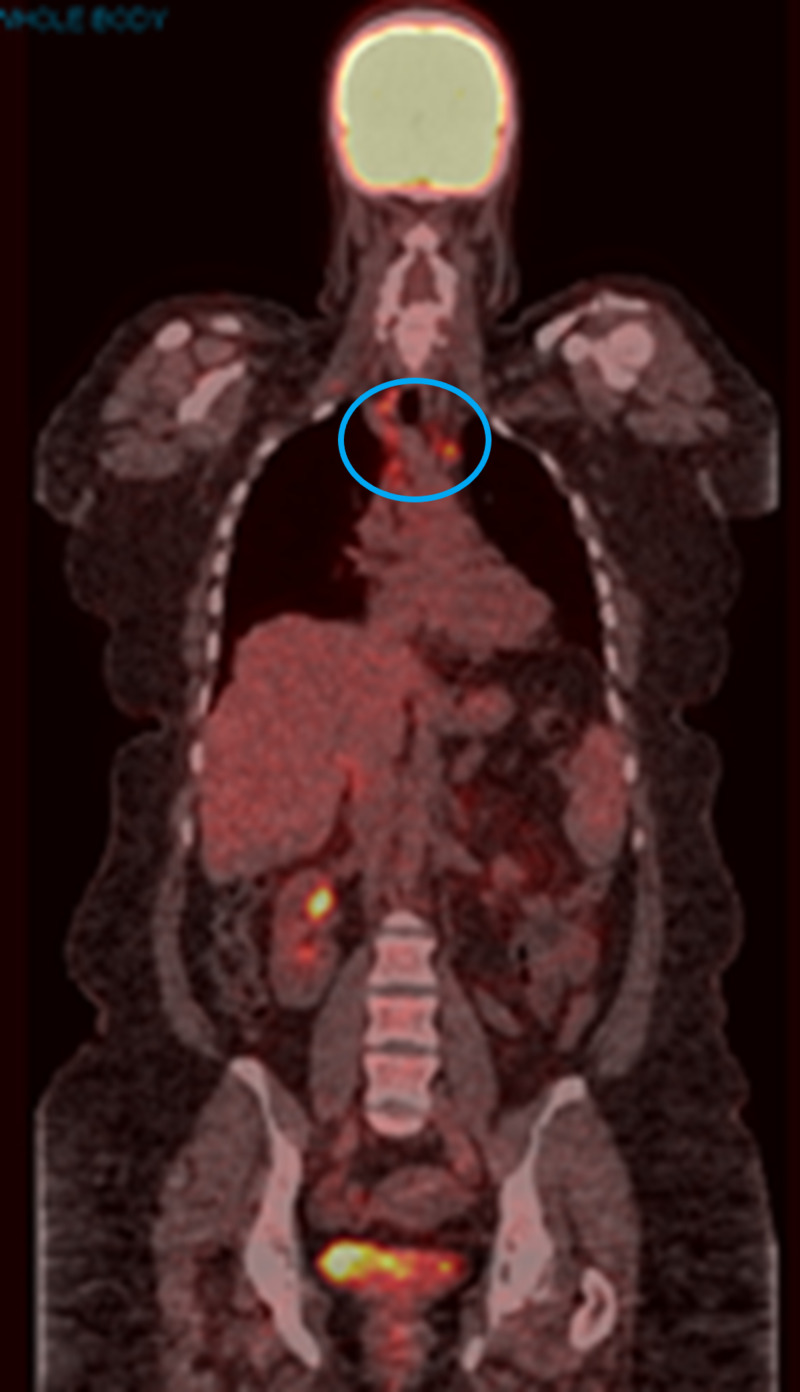
PET/CT scan in November 2018 The image shows persistent mediastinal thoracic adenopathy with increased uptake (blue circle) PET: positron emission tomography; CT: computed tomography

The patient continued to tolerate treatment well except for the occurrence of mild to moderate arthralgia, requiring intermittent short courses of steroids. Imaging remained stable until November 2018, when there was evidence of “progression.” PET/CT scan in November 2018 showed persistent mediastinal thoracic and abdominal adenopathy with increased uptake since the prior scan (Figure 4). The FDG uptake in subcarinal node measuring 1 cm increased from SUVmax 6.9 to 13.6. Before changing treatment, a decision was made to re-biopsy the FDG-avid mediastinal lymph nodes. Pathology from multiple mediastinal lymph nodes obtained by mediastinoscopy showed multiple lymph nodes that were negative for malignancy but revealed necrotizing granulomas; fungal and acid-fast bacilli (AFB) cultures were negative. After two years of treatment without evidence of progression, pembrolizumab was discontinued in April 2019. The patent continues to have surveillance visits and imaging. Serial PET/CT scans have shown multiple FDG-avid hilar, mediastinal, and periportal lymph nodes. She denies any cough or shortness of breath. Currently, she is also being followed up by rheumatology for arthralgia in ankles, wrists, and hands, which is partially relieved with steroids.

## Discussion

It can be hypothesized that in our patient, pembrolizumab caused the resolution of melanoma-associated FDG-avid lymphadenopathy within the first two months of starting the treatment. During this time, however, the patient developed transient hepatitis, vitiligo, and rash, all of which are known irAEs. Subsequent PET/CT scans showed the emergence of FDG-avid lymphadenopathy caused by granulomatous inflammation. 

The development of sarcoidosis or SLR during treatment of melanoma with immunotherapy is now well documented. Sarcoidosis most commonly presents with dyspnea, wheezing, chest pain, or a nonproductive cough [9]. The disease can also present with extrapulmonary symptoms ranging from general fatigue and weight loss to symptoms involving the skin, joints, liver, GI tract, and the heart [10]. Hypercalcemia, elevated liver enzymes, lymphopenia, elevated angiotensin-converting enzyme, and hypergammaglobulinemia have all been associated with an increased likelihood of sarcoidosis [9,10]. There is no definitive test for diagnosing the condition; the diagnosis of sarcoidosis requires a combination of clinical features, imaging characteristics, and histopathologic confirmation of granulomas [11,12].

## Conclusions

This case highlights the perils of interpreting FDG-PET scans during the treatment of melanoma with immunotherapy. Attributing all FDG-avid lymphadenopathy to malignancy can lead to premature termination of effective treatment and delayed diagnosis of inflammatory conditions. This highlights the importance of maintaining a broad differential diagnosis when interpreting diagnostic imaging to monitor treatment response. SLR should be considered in the differential diagnosis of PET-avid lymphadenopathy. In the appropriate setting, apparent radiologic progression should be confirmed with pathologic sampling before changing treatment. As we learned from this case, uptake on PET/CT scan is not always from cancer!
